# Expression and Promoter Analysis of Six Heat Stress-Inducible Genes in Rice

**DOI:** 10.1155/2013/397401

**Published:** 2013-12-26

**Authors:** Wirat Rerksiri, Xianwen Zhang, Hairong Xiong, Xinbo Chen

**Affiliations:** ^1^Key Laboratory for Crop Germplasm Innovation and Utilization of Hunan Province, Hunan Agricultural University, Changsha 410128, China; ^2^College of Bioscience and Biotechnology, Hunan Agricultural University, Changsha 410128, China; ^3^Center of Analysis and Testing, Hunan Agricultural University, Changsha 410128, China

## Abstract

During the long evolutionary process, plant gradually formed a series of strategies and mechanisms to cope with stress environment such as drought, heat, cold, and high salinity. Six highly heat responsive genes were identified in rice by microarray data analysis. The qRT-PCR analysis confirmed that the expression of these six genes were highly heat inducible and moderately responded to salt stress, polyethylene glycol, and abscisic acid treatment, but little affected by cold treatment. Promoters of the three highly heat-inducible genes (OsHsfB2cp, PM19p, and Hsp90p) were used to drive GUS gene expression in rice. The results of the GUS gene expression, histochemical staining, and GUS activities in panicles and flag leaves of the transgenic rice plants confirmed high heat-induced GUS activities and moderate drought-induced activities. The three promoters exhibited similar high activity lever in rice leaf under heat, but OsHsfB2cp and PM19p showed much higher activities in panicles under heat stress. Our work confirmed that the OsHsfB2c and PM19 promoters were highly heat inducible and further characterization and reconstruction of *cis*-elements in their promoters could lead to the development of highly effective heat-inducible promoters for plant genetic engineering.

## 1. Introduction

Rice is the most important staple food in Asia. More than 90% of the world's rice is grown and consumed in Asia, where 60% of the world's population lives. Rice plants are sensitive to various abiotic stresses [1]. As such, rice carries an odd portfolio of tolerances and susceptibilities to abiotic stresses as compared to other crops [2]. However, many rice-growing environments demand still greater tolerance than what has been found in most improved germplasm [3]. Global warming has been likely to induce heat stress more frequently and severely, resulting in serious yield reduction and thus threatening the security of grain production. Abiotic stresses such as high temperature or drought can induce diverse physiological and molecular responses in plants. These include extensive cellular damage and inhibition of photosynthesis, osmotic adjustment, induction of repair systems and chaperones, and changes of gene expression and metabolism [4]. Functional characterization of stress-inducible genes is important not only for understanding the molecular mechanisms of stress tolerance, but also for practical application in improving stress tolerance of crops by gene manipulation. To improve stress tolerance of crop plants, stress tolerant/resistant genes can be overexpressed in transgenic plants, using either constitutive or stress-inducible promoters. Although constitutive expression of stress resistant genes can improve resistance of transgenic plants to abiotic stresses, it may also cause stunted growth and reduction of yield in plants. Promoters that were subject to specific regulations are useful for manipulating foreign gene expression in plant cells, tissues, or organs with desirable patterns and under controlled conditions [5]. Therefore, inducible promoters including chemical inducible promoters, which drive gene expression only when plants are exposed to stresses or bioreactors to adapt the environment and maintain normal growth, are of great importance in biotechnology, molecular breeding, and agriculture [6].

In the present study, we have identified six abiotic stress responsive genes in rice from microarray data and further confirmed the expression pattern of these six genes in different organs and under abiotic stresses in rice by quantitative real-time PCR (qRT-PCR). Promoters of three highly heat-inducible genes (OsHsfB2cp, PM19p, and Hsp90p) were used to drive GUS gene expression, and the expression in transgenic rice panicles and flag leaves under heat and drought treatments was performed. These data and further work to come would be valuable for developing stress-inducible promoters in application.

## 2. Materials and Methods

### 2.1. Identification of Candidate Stress Inducible Genes from Microarray Data

In our previous study, the genome-wide gene expression analysis in response to heat shock was performed in rice panicle [7] and flag leaf [8] and the original microarray data were deposited in GEO database under accession numbers GSE38665 and GSE45259. Expression of six heat-responsive genes was derived from the above microarray data. The expression data of the six heat-responsive genes in rice seedlings under abiotic stresses were collected through the GEO accession numbers GSE14275 [9] and GSE6901 [10]. The six candidate genes in this study were named OsHsfA2a (Os03g0745000), Dehydrin (Os11g0453900), DnaJ protein putative (Os06g0195800), OsHsfB2c (Os09g0526600), AWPM-19 (Os05g0381400), and Hsp90 (Os04g0107900).

### 2.2. Plants Materials, Growth Condition, and Treatments

Rice seeds (*Oryza sativa* L. ssp. japonica) were imbibed in water in the dark at 28°C for 3 days. Germinated seeds were planted in plastic pots and grown in a climate chamber (Binder, Tuttlingen, Germany) at 25°C and 80% RH (relative humidity) with a 12 h light/12 h dark cycle and irrigated with 1/2 MS (Murashige and Skoog medium) liquid culture. After three weeks, the seedlings were subject to the abiotic stress treatments. For salt, polyethylene glycol (PEG), and abscisic acid (ABA) treatments, seedlings were irrigated with 1/2 MS liquid culture containing 100 mmol L^−1^ NaCl, 10% PEG, and 100 *μ*mol L^−1^ ABA, respectively. For cold and heat shock treatments, seedlings were exposed to 4°C and 42°C, respectively, and irrigated with 1/2 MS liquid culture. After stress treatment, samples were collected separately at 0 min, 30 min, and 1, 2, 4, 8, and 12 h. For tissue specific expression analysis, roots were obtained from the three-week-old rice seedlings grown under the same condition as the controls described in the stress treatment section. Stems, leaves, sheaths, and spikes were obtained from field grown rice plants at booting stage. Rice embryos were isolated from 48 h imbibed seeds. Three replications for each experiment were performed in this study. All samples were flash-frozen in liquid nitrogen and stored at −80°C for subsequent RNA extraction.

### 2.3. RNA Isolation and qRT-PCR Analysis

Total RNA was extracted using TRIZOL reagent (Invitrogen, Carlsbad, CA, USA), according to the manufacturer's protocol. The RNA samples were quantified by absorbance at 260 nm and by electrophoresis on a 1% agarose gel stained with ethidium bromide. Five micrograms of each RNA sample was incubated with 1 U DNase I (Fermentas, Vilnius, Lithuania) for 30 min at 37°C to remove residual DNA contamination. DNase I-treated RNA samples were then reverse transcribed with Oligo (dT)_18_ primers using ReverTra Ace (Toyobo, Osaka, Japan) for 60 min at 42°C. The cDNA samples were then diluted threefold with buffer (10 mmol L^−1^ Tris-HCl, pH 8.0, 1 mmol L^−1^ EDTA).

qRT-PCR was performed on an ABI 7300 real-time PCR system (Applied Biosystems) using SYBR Premix Ex Taq II (Takara, Tokyo, Japan) under the following conditions: 95°C for 30 s, followed by 40 cycles of 95°C for 5 s and 60°C for 31 s. A rice actin gene (*RAc1*, accession number AB047313) expression level was used as a positive internal control (Table 1). Three biological replicates and three technical replicates for each experiment were performed.

Tissue-specific expression of the six genes were analyzed using 2^Δ*C*_*T*_^, where Δ*C*_*T*_ = *C*_*T*_(Target) − C_T_  (Actin). The relative expression change of six genes in response to abiotic stresses was quantified using the 2^−ΔΔ*C*_*T*_^ method [11]. The data were expressed as mean ± standard error.

### 2.4. Bioinformatics Analysis of the Three Inducible Promoters

The 1.5 kb promoter sequences upstream of the start codon ATG of the 3 stress-inducible genes were extracted from the GenBank rice genome database, and promoter *cis*-elements were analyzed using PLANTCARE (http://bioinformatics.psb.ugent.be/webtools/plantcare/html/) and PLACE (http://www.dna.affrc.go.jp/PLACE/).

### 2.5. Plasmid Construction for the Three Stress-Inducible Promoters

The promoters of choice were amplified by PCR using promoter-specific primers (Table 2). The PCR was performed at 95°C for 2 min, followed by 36 cycles of 94°C for 30 s, 60°C for 30 s, and 72°C for 1 min, with a final cycle at 72°C for 8 min using 100 ng of genomic DNA. PCR products were detected by 1% agarose gel electrophoresis. The target fragments were recovered by DNA purification kit and ligated to the vector pMD19-T, and the product was transformed into *Escherichia coli* DH5*α*. Positive recombinant clones were selected by PCR and sequenced by Nanjing Jin Site Biotechnology Company. To construct the recombinant expression vectors for *Agrobacterium tumefaciens*, the pCAMBIA1301 vector was first digested with *Hind*III*/Bgl*II to remove the 35 S promoter sequence, then end-filled, and ligated as a circular vector. The target promoter fragments in the pMD19-T vectors were digested by *BamH*I*/Pst*I and inserted into the *BamH*I*/Pst*I sites of the modified pCAMBIA1301 vector to get the specific promoter-GUS recombinant plasmids

### 2.6. Plant Transformation

The promoter-GUS recombinant plasmids were mobilized into *Agrobacterium tumefaciens* LBA4404 using the freeze-thaw method [12]. Embryogeniccalli of Nipponbare were grown and transformed with *Agrobacterium* [13]. Transgenic calli were selected on selection medium consisting of MS salts and vitamins, 30 g/L sucrose, 2 mg/L 2,4-D, 3 g/L phytagel (P8169, Sigma-Aldrich) supplemented with 50 mg/L hygromycin B for 30 days with one transfer to fresh medium after 15 days. Transgenic plants were regenerated under hygromycin selection on regeneration medium (MS, 30 g/L sucrose, 2 mg/L kinetin, 0.5 mg/L NAA, 3 g/L phytagel). The regenerated plants were allowed to set roots for 10 days in rooting medium (one-half strength MS, 2 g/L phytagel) and followed by growing in water for 10 days before being transferred to pots in the greenhouse and finally grown to maturity. All plants were fertile with normal phenotype. The T_0_ transgenic rice plants grown in the greenhouse were confirmed for the presence of *gusA*. Subsequently, T_1_ and T_2_ generation of transgenic plants were raised. About 30 T_0_ transgenic plants for each of the promoter constructs were derived and grown up to T_2_ generation. At least three T_2_ transgenic lines for each promoter were considered for further characterization.

### 2.7. Heat and Drought Treatments on Transgenic Rice Lines

To evaluate the GUS gene expression and GUS activity in the promoter: GUS transgenic rice, heat, and drought stresses were performed at the reproductive stage. For heat stress treatment, whole plants were exposed to 40°C for 8 h. For drought treatment, the plants were withheld from water supply for 3 days until the leaves became rolled. The control plants were kept at 30°C and fully watered. The untreated plants were grown at normal conditions. The flag leaves and panicles were sampled and flash-frozen in liquid nitrogen and stored at −80°C. Three replications are performed for each experiment in this study.

### 2.8. GUS Gene Expression Analysis by qRT-PCR

The qRT-PCR was performed with *gusA* specific primers under the following conditions: 95°C for 30 s, followed by 40 cycles at 95°C for 5 s, and 60°C for 31 s using GUS5A (5′CGACGCTCACACCGATACCATC3′) and GUS3 (5′TCTCCTGCCAGGCCAGAAGTTC3′). The quantitative variation between different samples was evaluated. The amplification of rice actin gene (*RAc1*) using primers AF (5′CTTCAACACCCCTGCTATG3′) and AR (5′TCCATCAGGAAGCTCGTAG3′) was performed as internal control to normalize all data. The mean values for the expression levels of the target genes were calculated from three independent experiments. The relative expression level was calculated as 2^−ΔΔ*C*_*T*_^.

### 2.9. GUS Histochemical Staining

GUS histochemical staining was performed as described by Jefferson et al. [14]. The GUS reaction mixture consisted of the solution containing sodium phosphate (Na_4_PO_4_, pH 7.0), Triton-X-100, potassium ferricyanide (K_3_FeCN_6_), potassium ferrocyanide (K_4_FeCN_6_), and 5-bromo-4-chloro-3-indolyl-*β*-D-glucuronide (X-gluc). GUS histochemical staining on the spikelets and flag leaves from the three transgenic rice lines was conducted under heat and drought stresses. The samples were incubated in the solution at 37°C for 24 h. The reaction was stopped by adding 70% (v/v) ethanol and the pigments and chlorophylls were removed by repeated ethanol treatment.

### 2.10. Quantitative GUS Enzyme Activity Assay

Fluorometric assay for GUS enzyme activity was carried out according to the method of Jefferson et al. [14]. The extracted proteins were mixed with GUS assay buffer (2 mM 4-methylumbelliferyl-D-glucuronide (4-MUG), 50 mM sodium phosphate buffer with pH 7.0, 10 mM *β*-mercaptoethanol, 10 mM Na_2_EDTA, 0.1% sodium lauroylsarcosine, and 0.1% Triton X-100). The addition of the stop buffer (0.2 M Na_2_CO_3_) halted the reaction. Next, 4-MUG was hydrolyzed by GUS to produce 4-methylumbelliferone fluorochrome (4-MU). GUS activity was determined in triplicate with a microplate spectrofluorometer (Megellan, Taken). The excitation wavelength was 365 nm, and the emission wavelength was 455 nm.

## 3. Results 

### 3.1. Expression of Six Selected Rice Genes under Abiotic Stresses as Revealed by Microarray Data

**Figure 1:**
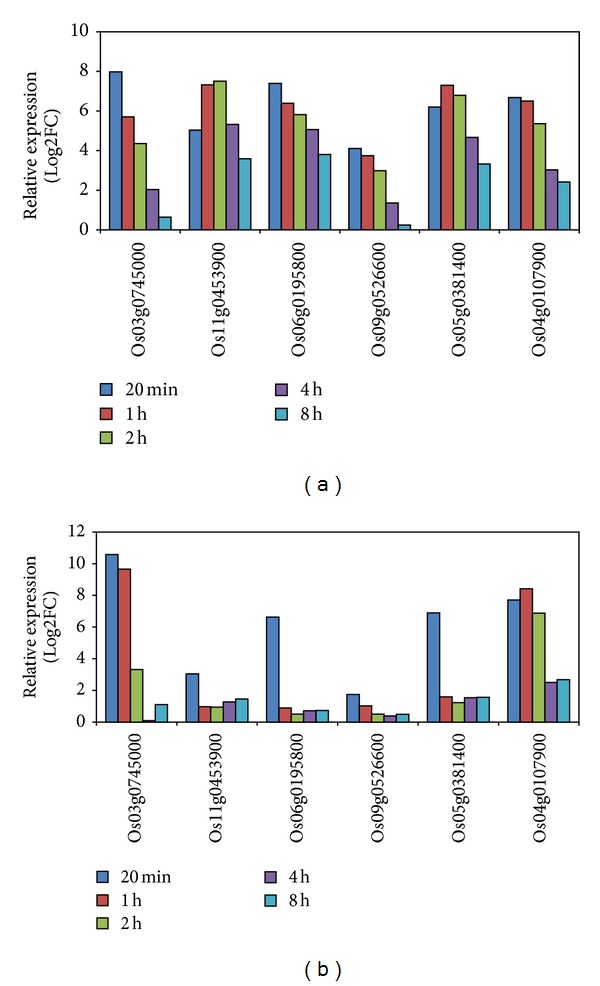
Expression of the six candidate genes in leaves and panicles under high temperature. (a) Panicle and (b) leaf. Log2FC: log2 transformed fold change. The expression data in panicle and leaf were obtained from GEO under accession numbers GSE38665 and GSE45259, respectively.

**Figure 2:**
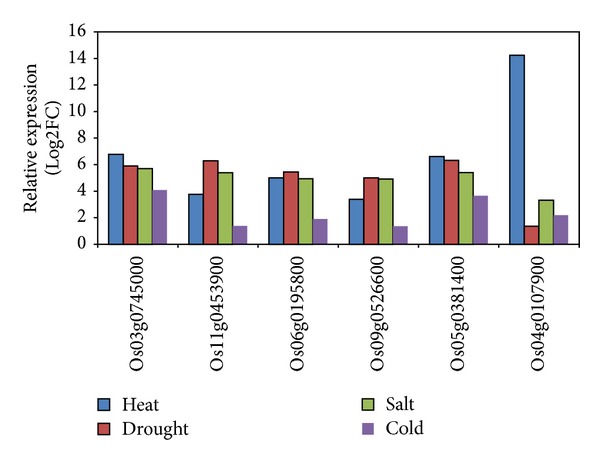
Expression of the six candidate genes in rice seedlings under different abiotic stresses. Log2FC: log2 transformed fold change. The expression data in rice seedlings under heat, drought, salt, and cold stresses were collected from GEO under the accession numbers GSE14275 and GSE6901, respectively.

Six genes (Os03g0745000, Os11g0453900, Os06g0195800, Os09g0526600, Os05g0381400, and Os04g0107900) were identified as highly heat-responsive genes from the microarray data of rice panicles under heat shock (Figure 1(a)). Our further microarray analysis in rice flag leaves under heat stress also confirmed that these six genes were highly heat inducible, although the expression levels decreased more quickly on time course treatment in flag leaves (Figure 1(b)). Microarray data of these six genes in rice seedling collected through the GEO accession numbers GSE14275 and GSE6901 showed that five of them were highly induced by heat, drought, and salt but less sensitive to cold stress, with the exception that Os04g0107900 was extremely heat inducible but insensitive to drought stress (Figure 2).

### 3.2. Expression Pattern of the Six Selected Genes in Different Tissues

**Figure 3:**
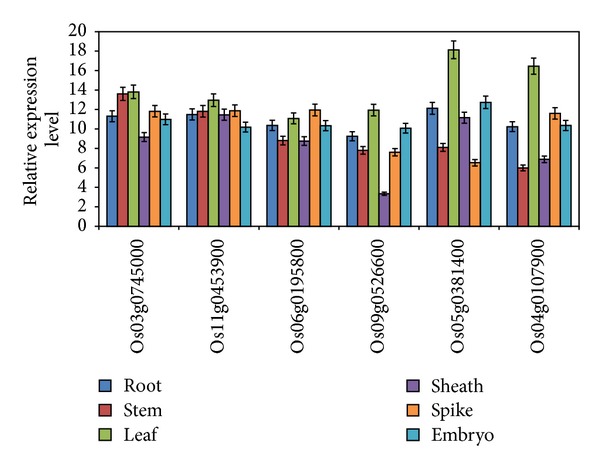
Tissue-specific expression analysis on the six genes by qRT-PCR. Error bars in the figures indicate standard deviation.

To examine the expression pattern of the six genes in different tissues, total RNA from the roots, stems, leaves, leaf sheath, spikes, and embryos were extracted. The results of qRT-PCR analysis showed that the six genes were expressed in all the organs under normal conditions. All of the genes were expressed at higher level in all organs, with the exception that the expression of Os09g0526600 was much lower in leaf sheath (Figure 3).

### 3.3. Expression Patterns of the Six Genes under Abiotic Stresses and ABA Treatment as Validated by qRT-PCR

**Figure 4:**
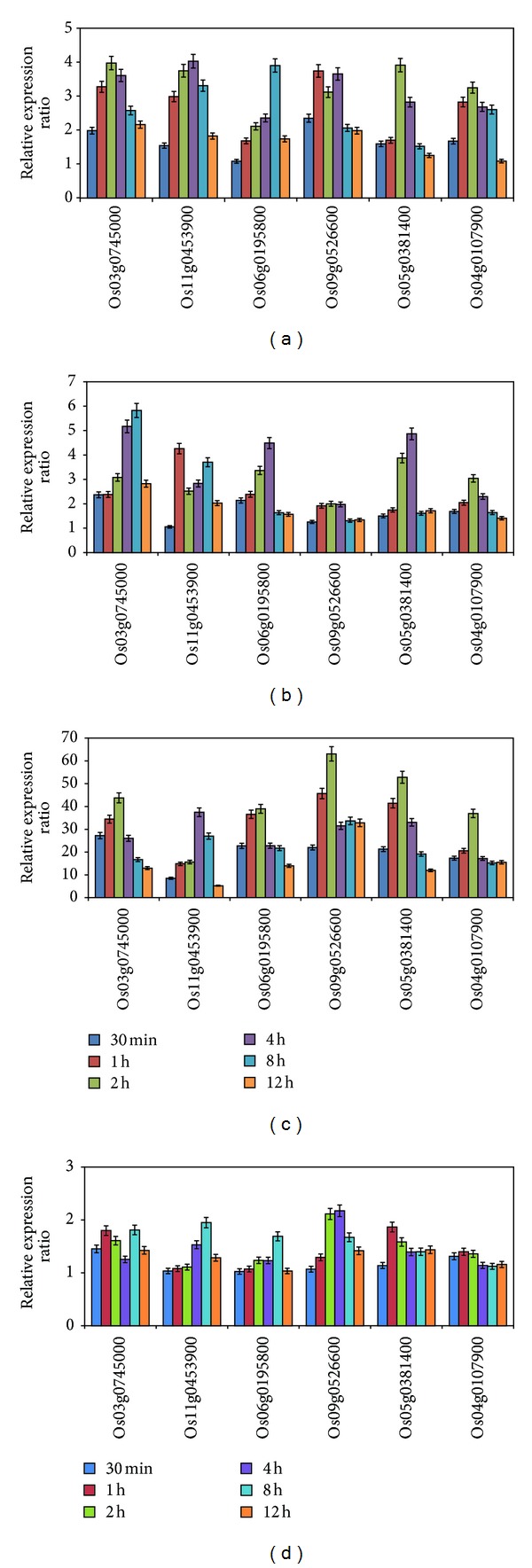
Expression confirmation of six genes under abiotic stresses by qRT-PCR. Three-week-old rice seedlings were subject to salt (100 mmol L^−1^ NaCl) (a), PEG treatment (10% PEG) (b), heat shock (42°C) (c), and cold treatment (4°C) (d). Error bars in the figures indicate standard deviation.

**Figure 5:**
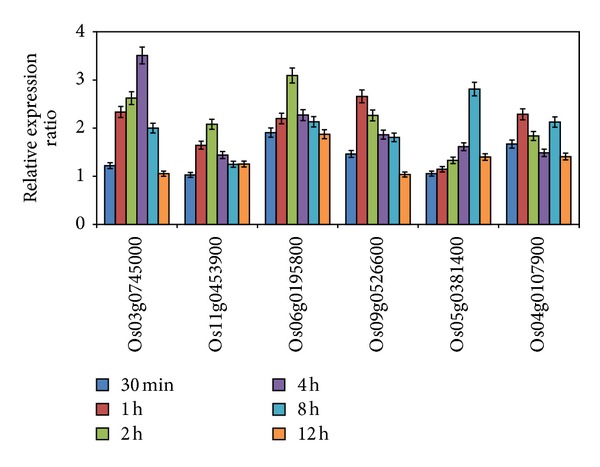
Expression profiles of the six genes in response to ABA treatment. Three-week-old rice seedlings were subject to 100 mmol L^−1^ ABA. Error bars in the figures indicate standard deviation.

To validate the stress induced expression level of the six genes obtained from the above microarray data, expression patterns of these six genes under different abiotic stresses were further analyzed by qRT-PCR. The expression of the six genes was induced by 100 mmol L^−1^ NaCl at 30 min and reached the highest level at 2 or 4 h and then began to decrease, except for the Os06g0195800 that reached the highest level at 8 h (Figure 4(a)). The expression of six genes was early upregulated by PEG treatment (Figure 4(b)). Compared to the responses to salt and PEG stresses, the expression of the six genes was rapidly induced to much higher level by heat treatment at 30 min (Figure 4(c)) along with 8.5-fold increase for Os11g0453900 and around 20-fold increase for the other five genes. The highest levels of these genes under heat stress were between 63- and 45-fold, which are much higher than the highest levels under salt and PEG stresses (less than 7-fold). The induced expression of Os09g0526600 and Os05g0381400 remained relatively steady under heat stress. The expression of the six genes was less affected by cold treatment with the highest expression of around 2-fold (Figure 4(d)). In addition, expression of all the six genes was affected by ABA to some extent (Figure 5).

### 3.4. Bioinformatics Analysis on the Promoters of Three Stress-Inducible Genes

**Figure 6:**
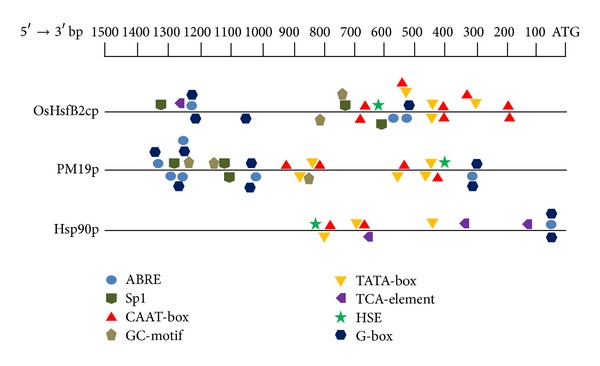
Bioinformatics analysis on the three stress-inducible promoters. The 1.5 kb promoter sequences upstream of the start codon ATG of the 3 stress-inducible genes were extracted from the GenBank rice genome database, and promoter *cis*-elements were analyzed using PLANTCARE (http://bioinformatics.psb.ugent.be/webtools/plantcare/html/) and PLACE (http://www.dna.affrc.go.jp/PLACE/).

Three highly stress-inducible genes (Os09g0526600, Os05g0381400, and Os04g0107900) were selected for promoter element and promoter-driven GUS expression analysis. Approximately 1.2 to 1.5 kb of the upstream region of the translation initiation codon (ATG) were predicted to be the promoter region of the stress-inducible genes and named OsHsfB2c (1220 bp), PM19 (1218 bp), and HSP90 (1437 bp), respectively. The promoter analysis by PlantCARE and PLACE showed some stress response-related elements, such as G-box, GC-motif, HSE, and ABRE in the three promoters (Figure 6).

### 3.5. Expression of GUS Gene Driven by the Three Stress-Inducible Promoters in Transgenic Rice

**Figure 7:**
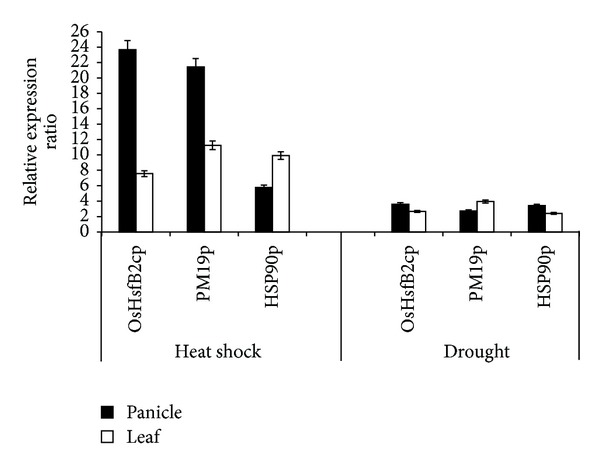
Detection of the GUS gene expression in transgenic rice lines by qRT-PCR. Error bars in the figures indicate standard deviation.

**Figure 8:**
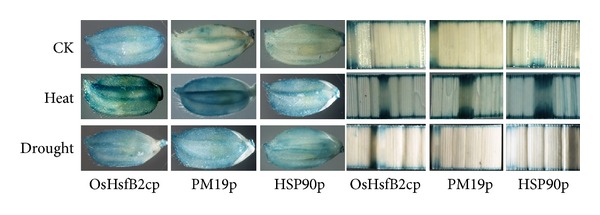
Histochemical staining of GUS activity in panicles and leaves of the transgenic rice lines.

**Figure 9:**
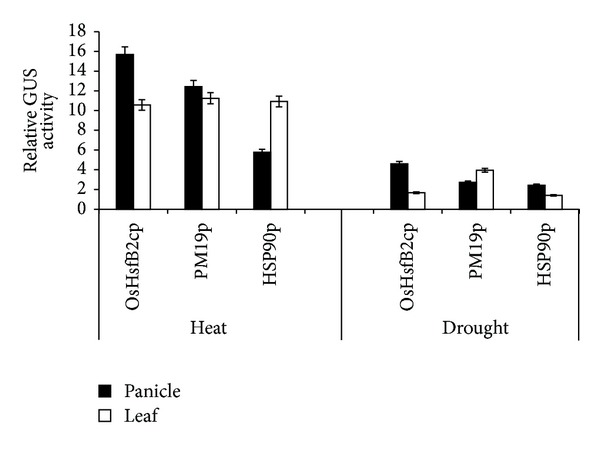
Quantitative fluorometric assay of GUS enzyme activity in transgenic rice lines under heat and drought stresses. Error bars in the figures indicate standard deviation.

The promoters of the three genes were fused to the *gusA* reporter gene to evaluate the heat and drought stress responses of these promoters in transgenic rice plants by monitoring the expression of *gusA* reporter gene and GUS enzyme activities. Total RNA was extracted from flag leaf and panicle in the presence or absence of drought and heat stress for qRT-PCR. The GUS gene expression levels significantly increased in both panicles and leaves for all the three genes under heat stress. In the leaves, the expression levels were upregulated around 8- to 11-fold. In panicles, the expression levels of OsHsfB2cp and PM19p were greatly increased to more than 20-fold, while the expression of Hsp90p was only increased about 6-fold. The GUS gene expression was less induced under drought both in panicles and leaves (Figure 7). GUS histochemical staining was prominently visualized in panicles and leaves in response to heat and drought stress (Figure 8). The three promoters showed a low activity in panicle and flag leaf under drought stress. Quantification of GUS activity using fluorescence assay demonstrated that the GUS activities driven by three promoters exhibited quite similar patterns under heat and drought stresses in both panicles and leaves of the transgenic rice lines (Figure 9). These data confirmed that three promoters of choice were highly heat inducible but weakly induced by drought treatment.

## 4. Discussion

Microarray has been widely used to systematically investigate the molecular reactions by which plants respond and adapt to complicated environment [15–17]. It was also applied to profile the tissue- and stress-specific gene expression [18–20], as well as transcriptome of rice developing caryopses under high temperature [21]. Based on our microarray data and rice abiotic stress microarray data available in the public GEO database, we identified six highly heat-inducible genes in rice for the promoter analysis with the aim to develop heat or abiotic stress inducible promoters for plant genetic engineering.

The transcripts of the six genes were accumulated in all the tissues we checked under normal condition. It has been elucidated that rice heat shock transcription factor (*OsHsf*) and heat shock proteins (*OsHsp*) were usually induced in response to abiotic stresses [22–29]. Os03g0745000 (*OsHsfA2a*) and Os09g0526600 (*OsHsfA2c*) were quickly and exceptionally highly induced under heat stress [22–24, 26]. *OsHsp*s were upregulated under heat, PEG, and ABA treatments [25, 29]. Our qRT-PCR analysis confirmed that the expression of six genes was induced under heat stress, moderately induced by salt and drought treatments, and less affected by cold treatment. High expression of abiotic stress tolerance genes in these important organs under stresses is a key requirement for a stress inducible promoter. These implied that the promoters of the selected genes may be useful for developing heat inducible promoters.

The GUS gene reporter system is one of the most effective techniques employed in the study of gene regulation in plant molecular biology [27]. In this study, we further evaluated the heat and drought stress responses of three promoters in panicle and flag leaf of the transgenic rice plants by qRT-PCR, histochemical staining, and fluorescence quantification. GUS gene expression and GUS protein activity in the leaves and panicles of the OsHsfB2cp and OsPM19p transgenic plants were significantly upregulated under heat stress but less induced under drought, which is supported by the bioinformatics analysis on the promoter sequences revealing that various stress-responsive elements such as HSE, ABRE, and G-box are located in the OsHsfB2cp and OsPM19p. These results further suggested that OsHsfB2cp and PM19p may be useful for developing heat inducible promoters.

High temperature can significantly affect various activities of plant. Leaf is the main organ for photosynthesis and is easily affected by heat stress. Reproduction organs are vulnerable to heat stress. Rice is most susceptible to heat injury during flowering, as pollen viability is particularly sensitive to heat stress; even 1-2 h of high temperature at anthesis would result in high spikelet sterility [22]. It was also reported that high temperature caused rice male sterility during pollen development [30]. A joint analysis of metabolome and transcriptome was performed to comprehend the impact of high temperature on rice grain filling [21]. The inducible expression of heat tolerant genes in reproductive organs will be helpful for rice and other crops to avoid production loss under heat stress. The present study showed that OsHsfB2cp and PM19p can drive high inducible gene expression in leave and panicles and may be useful for developing heat inducible promoters with further characterization and reconstruction of their *cis*-elements.

## 5. Conclusions

Plant growth and development, especially crop yield can be greatly affected by abiotic stresses. Global warming has been likely to induce heat stress more frequently and severely, resulting in serious yield reduction and thus threatening the security of grain production. Stress-inducible promoters can be used to drive specific gene expression under stresses for increased abiotic stress tolerance. Six highly heat responsive genes were identified from rice microarray data. Our qRT-PCR analysis confirmed that these six genes were highly heat inducible and moderately responsive to salt stress, PEG, and ABA treatments. Promoters of the three highly heat-inducible genes (OsHsfB2cp, PM19p, and Hsp90p) were used to drive GUS gene expression in rice. GUS gene expression, histochemical staining, and GUS activities analysis confirmed that OsHsfB2cp and PM19p were highly heat inducible in panicles and flag leaves. Our work suggests that OsHsfB2c and PM19 could be used as highly heat inducible promoters to drive specific gene expression under heat stress for increased stress tolerance.

## Figures and Tables

**Table 1:** Primer sequences of the 6 candidate genes for qRT-PCR.

Gene name	Forward primer (5′ to 3′)	Reverse primer (5′ to 3′)
Os03g0745000	GCGACACCGAGAGCTTCTGGATC	TCGTCATCCTCCTCGTCGTTGT
Os11g0453900	TCCAGCTCCAGCTCATCCTCT	GTCGTTGTTGTGGTGTGCTCCT
Os06g0195800	TGCGGAGAGCTTCGAGGAGTT	ACCGCTTGTTCACGCCCTTG
Os09g0526600	GTCCAGCTCCAGCCAAACGAT	CCTTACGCCACTACCGCATTCC
Os05g0381400	CGCTGCTGGTGCTGAATCTGAT	AGGATGGCGAAGACGAGGAAGT
Os04g0107900	ACGCCAAGGAGAGGAAGAGGTC	ATGTTCGCCGTCCAGCCGTA
RAc1	CTTCAACACCCCTGCTATG	TCCATCAGGAAGCTCGTAG

**Table 2:** Primers for cloning the 3 candidate promoters.

Promoters	Primer sequence (5′ to 3′)	Product size (bp)
OsHsfB2cp	Forward GGGTACCTATCAAAATCAATCTCAAAATTAAGCTTG	1220
	Reverse ACTGCAGGGCGAGGACCGACCGTAGTAG	

PM19p	Forward TAAGCTTGGCGCGTATCGACCATGGTT	1218
	Reverse CGGATCCGCAACACAATTAGCACTGGTTTAGCT	

Hsp90p	Forward CGGTACCTTTCTGATTGTATTTCTACGTGGACACT	1437
	Reverse ACTGCAGAATGTTGCCTGCTGCTTGAATG	

## References

[1] Gao J-P, Chao D-Y, Lin H-X (2007). Understanding abiotic stress tolerance mechanisms: recent studies on stress response in rice. *Journal of Integrative Plant Biology*.

[2] Lafitte HR, Ismail A, Bennet J (2004). Abiotic stress tolerance in rice for Asia: progress and the future, in new directions for a diverse planet. *Plant Cell*.

[3] Li SH, Tian XH, Huang YP, Liu AY (2007). Preliminary analysis of high temperature harm in middle-season rice at flowering stage in Jianghan Plain in the latest 50 years. *Chinese Journal of Agrometeorology*.

[4] Sarangi S, Ghosh J, Bora A, Das S, Mandal AB (2011). Agrobacterium-mediated genetic transformation of indica rice varieties involving Am-SOD gene. *Indian Journal of Biotechnology*.

[5] Yu S-M, Ko S-S, Hong C-Y (2007). Global functional analyses of rice promoters by genomics approaches. *Plant Molecular Biology*.

[6] Corrado G, Karali M (2009). Inducible gene expression systems and plant biotechnology. *Biotechnology Advances*.

[7] Zhang XW, Li JP, Liu AL (2012). Expression profile in rice panicle: insights into heat response mechanism at reproductive stage. *PLos ONE*.

[8] Zhang XW, Rerksiri W, Liu AL (2013). Transcriptome profile reveals heat response mechanism at molecular and metabolic levels in rice flag leaf. *Gene*.

[9] Hu W, Hu G, Han B (2009). Genome-wide survey and expression profiling of heat shock proteins and heat shock factors revealed overlapped and stress specific response under abiotic stresses in rice. *Plant Science*.

[10] Jain M, Nijhawan A, Arora R (2007). F-Box proteins in rice. Genome-wide analysis, classification, temporal and spatial gene expression during panicle and seed development, and regulation by light and abiotic stress. *Plant Physiology*.

[11] Livak KJ, Schmittgen TD (2001). Analysis of relative gene expression data using real-time quantitative PCR and the 2-ΔΔCT method. *Methods*.

[12] Hofgen R, Willmitzer L (1988). Storage of competent cells for Agrobacterium transformation. *Nucleic Acids Research*.

[13] Toki S, Hara N, Ono K (2006). Early infection of scutellum tissue with Agrobacterium allows high-speed transformation of rice. *Plant Journal*.

[14] Jefferson RA, Kavanagh TA, Bevan MW (1987). GUS fusions: beta-glucuronidase as a sensitive and versatile gene fusion marker in higher plants. *EMBO Journal*.

[15] Gonzali S, Loreti E, Novi G, Poggi A, Alpi A, Perata P (2005). The use of microarrays to study the anaerobic response in Arabidopsis. *Annals of Botany*.

[16] Brown PO, Botstein D (1999). Exploring the new world of the genome with DNA microarrays. *Nature Genetics*.

[17] Lockhart DJ, Winzeler EA (2000). Genomics, gene expression and DNA arrays. *Nature*.

[18] Fujita M, Horiuchi Y, Ueda Y (2010). Rice expression atlas in reproductive development. *Plant and Cell Physiology*.

[19] Wang L, Xie W, Chen Y (2010). A dynamic gene expression atlas covering the entire life cycle of rice. *Plant Journal*.

[20] Endo M, Tsuchiya T, Hamada K (2009). High temperatures cause male sterility in rice plants with transcriptional alterations during pollen development. *Plant and Cell Physiology*.

[21] Yamakawa H, Hakata M (2010). Atlas of rice grain filling-related metabolism under high temperature: joint analysis of metabolome and transcriptome demonstrated inhibition of starch accumulation and induction of amino acid accumulation. *Plant and Cell Physiology*.

[22] Jagadish SVK, Muthurajan R, Oane R (2010). Physiological and proteomic approaches to address heat tolerance during anthesis in rice (*Oryza sativa* L.). *Journal of Experimental Botany*.

[23] Chauhan H, Khurana N, Agarwal P, Khurana P (2011). Heat shock factors in rice (*Oryza sativa* L.): genome-wide expression analysis during reproductive development and abiotic stress. *Molecular Genetics and Genomics*.

[24] Singh A, Mittal D, Lavania D, Agarwal M, Mishra RC, Grover A (2012). OsHsfA2c and OsHsfB4b are involved in the transcriptional regulation of cytoplasmic *OsClpB (Hsp100)* gene in rice (*Oryza sativa* L.). *Cell Stress and Chaperones*.

[25] Ye SF, Yu SW, Shu LB, Wu JH, Wu AZ, Luo LJ (2012). Expression profile analysis of 9 heat shock protein genes throughout the life cycle and under abiotic stress in rice. *Chinese Science Bulletin*.

[26] Liu A-L, Zou J, Zhang X-W (2010). Expression profiles of class a rice heat shock transcription factor genes under abiotic stresses. *Journal of Plant Biology*.

[27] Fior S, Gerola PD (2009). Impact of ubiquitous inhibitors on the GUS gene reporter system: evidence from the model plants Arabidopsis, tobacco and rice and correction methods for quantitative assays of transgenic and endogenous GUS. *Plant Methods*.

[28] Chauhan H, Khurana N, Agarwal P, Khurana P (2011). Heat shock factors in rice (*Oryza sativa* L.): genome-wide expression analysis during reproductive development and abiotic stress. *Molecular Genetics and Genomics*.

[29] Zou J, Liu A, Chen X (2009). Expression analysis of nine rice heat shock protein genes under abiotic stresses and ABA treatment. *Journal of Plant Physiology*.

[30] Endo M, Tsuchiya T, Hamada K (2009). High temperatures cause male sterility in rice plants with transcriptional alterations during pollen development. *Plant and Cell Physiology*.

